# Correspondence

**Published:** 1899-02

**Authors:** 


### CORRESPONDENCE.

### INTERESTING CORRESPONDENCE.

MARSHALL CLARK CO ILL

tu tha Enquirer Cincinnati Sir Pleas Cipe this bafor tha Popplick tu Prevint Hog Collara Prepar yursalf with lime Sat it in yur barn lat aearslac Give tu yur hogs in slop or Wottar and Cipe yur Hogs from tacan Collara that Dissis is missiles give lime 2 or 3 tims a week or mix arSlac lime and salt lat yur Hogs lick it

Corrisspondis Pleese Coppy

H. C. YAHLBUSH, v.S.

---

BOZEMAN, MON., Dec. 27, 1898.

EDITOR JOURNAL OF COMPARATIVE MEDICINE, ETC.

A short time since there fell into my hands a little booklet of cures practised by a sort of witch doctor in Ohio, about fifty years ago. The booklet is written in low German, or "Pennsylvania Dutch." It contains in all one hundred formulas of remedies to be used or words to be spoken in a mystic way. The formulas may not be new to your readers, but I thought some of them of sufficient interest to translate and submit to you. The majority of the formulas are in an exceedingly quaint poetical form, but I have not dared to attempt a metrical version. Much of the force of the original is lost by my translation.

Very truly yours, E. V. WILCOX.

No. 6. For a strain. "You have sprained your leg. They hanged Christ on the cross. If his hanging did him no harm, your sprain does you no harm."

No. 7. When man or beast steps on a nail. "This is the nail with which Christ was nailed to the cross. Let there be no swelling or any other harm."

No. 9. For the lockjaw. "Hephada, hephada, hephada, open up!"

No. 10. For the flux. "We have only to pulverize a piece of red brick of the size of an egg from an oven, to dissolve it in fresh water and give it to the patient to drink cold."

No. 11. "In order to see of a night one should smear the eyes with bat's blood."

No. 13. To break a horse. "Brown horse or nag, be as gentle and patient for bridle, saddle, or harness as was Jesus Christ."

No. 18. To make anyone bring back stolen property. "Take three pieces of bread, three grains of salt, and three pieces of lard, and put them on a hot fire. Remain alone and say these words three times: 'I lay bread, salt, and lard on your fire, O thief! on account of your sin and your audacity. I put them upon your lungs, liver, and heart, so that pain and anxiety like a death-agony shall come upon you. All your veins shall be ruptured and death-pains will give you no peace until you return the stolen property to its place.'"

No. 21. To open a jewel-case. "Kill a tree-toad and expose it in the sun for three days. Reduce it to a powder. Put a little of the powder in the lock and it will open of itself."

No. 25. For tapeworms, threadworms, and hairworms. "The Lord God walked three furlongs in a goodly field. He found three worms—the struggleworm, the gnawworm, and the hairworm. Struggleworm, gnawworm, hairworm, go out of this flesh!"

No. 27. For fever. "Take a fresh-laid egg, boil it hard, take off the shell while hot, and repeat these words: 'Ben, aba, mahy, froha.' Eat it hot in three bites and then fast."

No. 28. For the flux. "Take a hard-boiled egg; eat it as hot as you can, without bread, salt, or anything else."

No. 33. For erysipelas. "Oh, you hottest burning carbuncle, how hot and red you are! With the help of the Trinity I have sought, found, and vanquished you!"

No. 36. To prevent mice from doing harm in the pantry. "When you put away the first loaf take it in the right hand and say: 'Here I lay away bread for man, but death to all mice and vermin.'"

No. 39. To cure boils. "Take a little anise-oil and turpentine; mix and put on the boil."

No. 40. To heal a severed vein. "Mash fishworms with old bacon. Put on warm.

No. 44. For suppression of the menses. "Boil grated Indian turnip in wine. Drink morning and evening."

No. 48. For sweney. "Sweney, disappear as the sun disappears from the sky."

No. 50. To cure worms. "As Job sat in the wilderness the horrid worms were about to devour him. 'O, God! hast thou forgotten me?' The worms shall not devour you. Whether they be black, white, green, yellow, or red, they shall quickly be dead, dead, dead."

No. 55. To cure cancer, scrofula, or anything. "You must arise before daybreak and without being spoken to must go to a thornbush and say: 'Good morning, Mr. Thornbush.' 'Good morning, sir. Why came you here?' 'I came here to cure (here name the case and the person).' Pull off a twig and throw it under the bush."

No. 60. For fever. "Cut into a cat's ear, let three drops of the blood fall into some brandy, add a little pepper and drink it."

No. 61. To cure a cough. "Roast a white onion and put it on the sole of the foot."

No. 62. To cure corns. "Find a red snail, rub the corn witq it, and carry the snail in your coat. The corn will disappear."

No. 70. To make the hair grow. "Take dog's milk and smear it on the spot."

No. 76. For incontinence of the urine. "Burn the hoof of a buck to powder and drink it in wine."

No. 92. To improve the memory. "Take the gall of a turkey and smear it on the temples near the ears."

---

Dr. H. D. Fenimore, of Knoxville, Tenn., received an appointment by the Bureau of Animal Industry, but did not accept it.

Drs. A. Drinkwater and J. C. McKenzie, of Rochester, N. Y., have tendered their resignations as veterinarians to the Humane Society of that city after many years of recognized and well-appreciated services by the officers and members of that organization.

Dr. E. B. Truitt, of Hillsboro, Ill., conducts a hospital in connection with his practice.

Dr. Harry Lukes, of Springfield, Mass., reports professional work on the increase through that territory.

Dr. John R. Mohler, of Milwaukee, Wis., Inspector in the Bureau of Animal Industry, was a visitor to his home in Philadelphia in January.

Dr. C. R. Jolly, of Atlanta, Ga., now conducts a hospital in connection with his practice in that city.

Dr. A. J. Tuxhill, of Auburn, N. Y., has entered one of the Empire State hospitals to undergo a surgical operation.

